# DeepEZ: A Graph Convolutional Network for Automated Epileptogenic Zone Localization From Resting-State fMRI Connectivity

**DOI:** 10.1109/TBME.2022.3187942

**Published:** 2022-12-26

**Authors:** Naresh Nandakumar, David Hsu, Raheel Ahmed, Archana Venkataraman

**Affiliations:** Department of Electrical and Computer Engineering, Johns Hopkins University, USA.; Department of Neurology, University of Wisconsin, USA.; Department of Neurosurgery, University of Wisconsin, USA.; Department of Electrical and Computer Engineering, Johns Hopkins University, Baltimore, MD 21218 USA

**Keywords:** Brain Connectivity, Deep Learning, Epilepsy, Seizure Localization, Resting-State fMRI

## Abstract

**Objective:**

Epileptogenic zone (EZ) localization is a crucial step during diagnostic work up and therapeutic planning in medication refractory epilepsy. In this paper, we present the first deep learning approach to localize the EZ based on resting-state fMRI (rs-fMRI) data.

**Methods:**

Our network, called DeepEZ, uses a cascade of graph convolutions that emphasize signal propagation along expected anatomical pathways. We also integrate domain-specific information, such as an asymmetry term on the predicted EZ and a learned subject-specific bias to mitigate environmental confounds.

**Results:**

We validate DeepEZ on rs-fMRI collected from 14 patients with focal epilepsy at the University of Wisconsin Madison. Using cross validation, we demonstrate that DeepEZ achieves consistently high EZ localization performance (Accuracy: 0.88 ± 0.03; AUC: 0.73 ± 0.03) that far outstripped any of the baseline methods. This performance is notable given the variability in EZ locations and scanner type across the cohort.

**Conclusion:**

Our results highlight the promise of using DeepEZ as an accurate and noninvasive therapeutic planning tool for medication refractory epilepsy.

**Significance:**

While prior work in EZ localization focused on identifying localized aberrant signatures, there is growing evidence that epileptic seizures affect inter-regional connectivity in the brain. DeepEZ allows clinicians to harness this information from noninvasive imaging that can easily be integrated into the existing clinical workflow.

## I. Introduction

Epilepsy is one of the most common neurological disorders, affecting around 50 million people worldwide, and is linked to a fivefold increase in mortality [13]. Epilepsy onset often occurs in childhood, and approximately one third of all patients have a medication refractory course that is associated with a disabling cumulative effect on neurocognitive development, lost productivity for the family, and increased societal and healthcare costs [5]. Surgical treatment is a safe and effective therapeutic approach for medication refractory epilepsy, that can provide seizure freedom and improved quality of life [26]. However, surgical candidacy and treatment outcomes are dependent on accurate localization of the epileptogenic zone (EZ) as defined by clinical, radiographic (magnetic resonance imaging, MRI) and physiological (electroencephalography, EEG) features [65]. Long-term treatment failures following surgery most commonly occur due to inaccurate identification and resection of the EZ. Invasive monitoring using implanted intracranial electrodes can provide more accurate EZ localization that can help plan treatment, but is associated with surgical risks [76]. Hence an accurate EZ localization hypothesis is the foundation for effective and safe treatment in epilepsy [45], [52], and is the most important prognostic determinant for long term treatment outcomes.

### A. Automated Methods for EZ Localization

Over the past two decades, there has been an increasing focus on automated methods for EZ localization. These methods are most often based on electrographic (EEG) or neuroimaging (structural MRI) modalities and can help reduce interpretative differences and delays in clinical reviews.

Automated methods for EEG localization have largely focused on improving the spatial resolution of the EEG sensors by deconvolving the signals into current dipoles or distributed sources at the millimeter scale [69], [84]. Going one step further, EEG data can be combined with noninvasive magnetoencephalography (MEG) for improved source estimation [58], [60]. Recent studies have demonstrated the translational promise of such methods. However, from a modeling standpoint, these *inverse solvers* require careful annotations of the seizure interval and are sensitive to physiological noise and the underlying head model [19], [20]. More importantly, they rely on high-density recordings of >50 EEG/MEG channels. From a logistical standpoint, the current standard-of-care for long-term EEG monitoring is the 10–20 electrode placement system [42], which contains fewer than 20 EEG channels distributed across the scalp. This resolution is insufficient for accurate and fine-grained inverse source estimation. Moreover, only 27% of epilepsy centers in the United States have access to and regularly utilize MEG [7]. Thus, while inverse source mapping remains a valuable direction of research with tremendous potential for presurgical evaluation, these methods are not amenable to most clinical workflows. Recently, Temple University Hospital (TUH) released a large public EEG dataset, which has spurred interest in seizure type classification [4], [66], [67], where the goal is to predict the epilepsy subtype from scalp EEG. While this task provides more information than seizure detection and is less reliant on human annotations than inverse source localization, the categories (focal, generalized, complex partial, absence, etc.) are too broad to accurately pinpoint the EZ.

In contrast to EEG, automated methods for MRI localization aim to identify epileptogenic lesions including Focal Cortical Dysplasias (FCDs), that are often difficult to radiographically identify on clinical imaging. Traditionally, these methods were implemented as a two-stage procedure. First, image-based features are extracted from the MRI data, such as cortical thickness, intensity, texture, asymmetry, and voxel-based morphometry [6], [16], [27], [34], [71]. Second, each voxel is classified as normal or FCD using statistical or machine learning algorithms. While these methods work well on large FCD cohorts, they tend to be unreliable for nonlesional patients [3]. In addition, epileptogenic lesions are diverse and can involve cortical, subcortical white matter [23], [63] and vascular abnormalities [24], [31], [78], which are better suited to other data modalities [2], [51], [81].

### B. Connectivity as a Biomarker for Epilepsy

Recent neuroimaging studies of epilepsy have implicated global brain network changes in seizure generation and disease progression. Accordingly, epilepsy is increasingly viewed as a network disorder that affects regional and global *connectivity* [10], [17], [38], [63], [68]. Diffusion MRI (d-MRI) assesses white matter properties based on free water diffusion [79] and informs us on the structure of brain networks [8]. In contrast, resting-state functional MRI (rs-fMRI) quantifies the temporal synchrony between brain regions by measuring changes in low frequency BOLD fluctuations. Alterations in structural and functional network properties have been linked to disease onset [25], duration [53], and treatment outcomes [10], [68] in epilepsy. For example, our group has investigated functional topology in subjects with epilepsy [30], demonstrating functional reorganization with a shift of network hubs to the contralateral hemisphere in temporal onset epilepsy [50]. Support Vector Machine-based analysis can discriminate network features in temporal epilepsy and healthy control subjects. In addition, neural connectivity patterns can help predict neuropsychological measures that assess language and memory function. Notably, global connectome changes in epilepsy are associated with a decrement in neurocognitive phenotype [72]. While promising, these connectivity studies are restricted to predefined structural and functional systems and careful patient subtyping (e.g., temporal lobe epilepsy). In addition, the results are correlative and do not quantify how well the biomarkers would generalize to new patients.

Recently, a few seminal studies have explored prospective EZ localization from rs-fMRI connectivity. The earliest work of [73] computed local and global network theoretic measures from whole-brain rs-fMRI data. The authors used outlying values in these network measures, as compared to a normative cohort, to define the EZ for each epilepsy patient. While promising on a small validation dataset, the performance requires careful tuning of different threshold values. The follow-up study of [75] proposed a hierarchical Bayesian model that inferred patient-specific hubs of abnormal connectivity. The authors demonstrate comparable performance to [73] without any auxiliary parameter tuning. However, both of these studies rely on comparison with a normative cohort, which may not be available in a clinical setting. The work of [85] takes a difference approach by first running independent component analysis (ICA) and the constructing a set of rules (e.g., asymmetry, power spectrum) to select the components associated with the EZ. While the authors demonstrate highly promising localization performance, they rely on visual inspection to select between candidate EZ components. Thus, a careful read of the method suggests that it is not fully automated. Specifically, the authors rely on visual inspection to select between candidate EZ components. Finally, the work of [36] also runs ICA on the rs-fMRI data and extracts a set of hand-crafted features from the components. In this case, the authors employ a support vector machine classifier to automatically learn which components are associated with the EZ. While the authors demonstrate good localization performance on some patients, the reported sensitivity is low for others.

### C. Our Contributions

In this paper, we introduce the first deep learning model for EZ localization using interictal rs-fMRI connectivity. The underlying assumption of our work is that the chronic and recurrent seizure activity causes subtle and distributed changes in functional connectivity across the brain. In contrast to rule-based approaches, we used supervised learning to automatically mine and leverage complex relationships in the rs-fMRI data for robust and generalizable EZ identification. Our model, which we call DeepEZ, takes as input a whole-brain connectivity graph, where nodes correspond to regions in our brain parcellation and edges denote the functional connectivity between regions. From here, DeepEZ uses graph convolutional networks (GCNs) [41], [83] to implicitly track information flow along expected anatomical pathways and fully-connected layers to classify each node (i.e., region) as belonging to the EZ or not. We encode anatomical information using d-MRI tractography, which is often viewed as the anatomical substrate for functional signaling in the brain [33], [82]. DeepEZ also incorporates the findings of previous works via an asymmetry term in the loss function to encourage lateralized predictions and a learned subject-specific bias to mitigate environmental confounds. We validate DeepEZ on a heterogeneous dataset of 14 pediatric epilepsy patients collected at the University of Wisconsin (UW) Madison. We demonstrate the DeepEZ outperforms the ICA methods of [36], [85] and ablated versions of the network. We also rigorously evaluate the sensitivity of DeepEZ to parcellation size, the number of network layers, hyperparameter tuning, and data augmentation. Taken together, our results highlight the promise of using rs-fMRI connectivity as a complementary source of information to localize the EZ in presurgical epilepsy patients.

## II. Materials and Methods

### A. Data and Preprocessing

Our dataset consists of preoperative functional and postoperative structural MRI scans from 14 pediatric subjects with focal epilepsy that underwent a EZ resection procedure at UW Madison. The MRI data was acquired as a part of standard care on either a GE 1.5 T or a GE 3 T Signa scanner. This study was approved by the University of Wisconsin-Madison Institutional Review Board under protocol 2019–1265 (approved Feb 2020).

Preoperative Rs-MRI (rs-fMRI) data was acquired using an echo planar imaging sequence (EPI, TR = 802 ms, TE = 33.5 ms, flip angle = 50°, FOV = 20.8 cm, res = 2 mm isotropic). The data was preprocessed using the CPAC pipeline, which includes slice time correction, motion correction, nuisance signal regression, band-pass filtering (0.01 – 0.1*Hz*), and registration to the MNI template. We use the Brainnetomme atlas [29] to define *N* = 246 cortical and subcortical regions for our analysis. We chose this atlas due to its fine spatial resolution and symmetric region definitions.

**Fig. 1.**
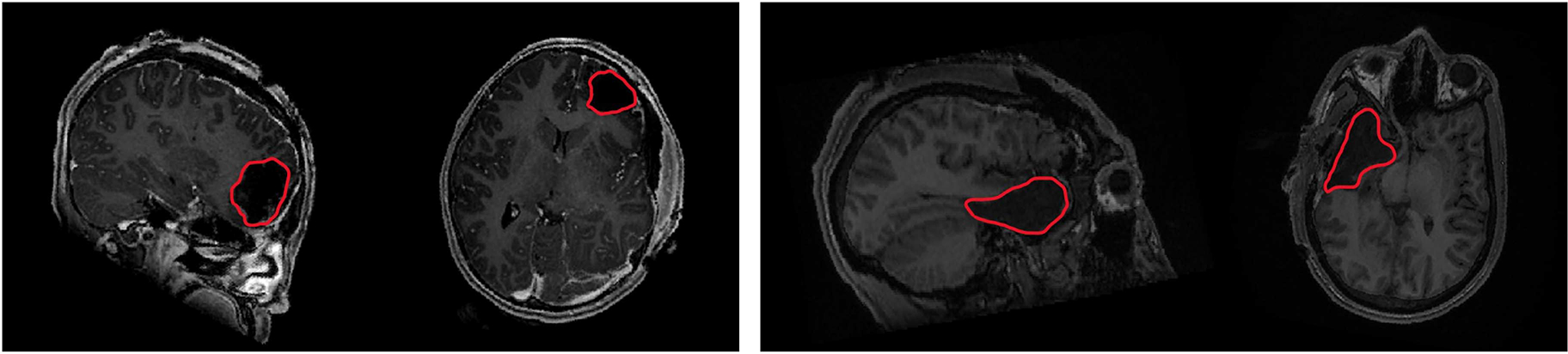
From (**L-R**), post resection structural MRI scans of two separate patients. We use the resection boundary, delineated via the red lines above, to derive pseudo ground truth labels for the EZ during training and testing.

Postoperative T1-weighted structural images were acquired using a three-dimensional gradient-echo pulse sequence (MPRAGE, TR= 604ms, TE= 2.516ms,flipangle= 8°, FOV = 25.6 cm, res = 0.8 mm isotropic). After skull stripping, we use affine registration to align the T1 data for each patient to the MNI space. All registrations were visually inspected for quality assurance. We manually delineate the resection zone and use this boundary to define pseudo ground truth EZ labels for training and evaluation after applying the Brainnetomme atlas. Fig. 1 depicts two examples of the post-operative T1 images, where the resection is marked by red arrows. Finally, Table I reports the age, gender, EZ location, scanner used to acquire the rs-fMRI data for each patient and patient outcome using the Engel [28] and ILAE scale [9]. As seen, our epilepsy cohort is highly heterogeneous and every patient experienced reduced seizures after surgery, with the majority being completely seizure free.

The GCNs in DeepEZ rely on a structural connectivity profile. To ensure consistency across subjects, we derive the graph from d-MRI tractography of 50 subjects from the Human Connectome Project (HCP) dataset [80]. The d-MRI data for each subject was preprocessed using the pipeline of [44] to obtain individual structural connectivity matrices based on the Brainnetome atlas. The steps of the pipeline include linear registration, tensor estimation, tractography, and graph estimation. The graphs are then averaged and thresholded to obtain the template matrix **A** used in the following section. Diffusion MRI (d-MRI) was also acquired for each patient and integrated into one of the baseline algorithms. D-MRI was collected on a 3 T GE scanner (TR= 7000ms, TE= 82.4ms, res= 1.5 mm isotropic, b-value = 1000).

### B. Deep Learning for EZ Localization

**Fig. 2.**
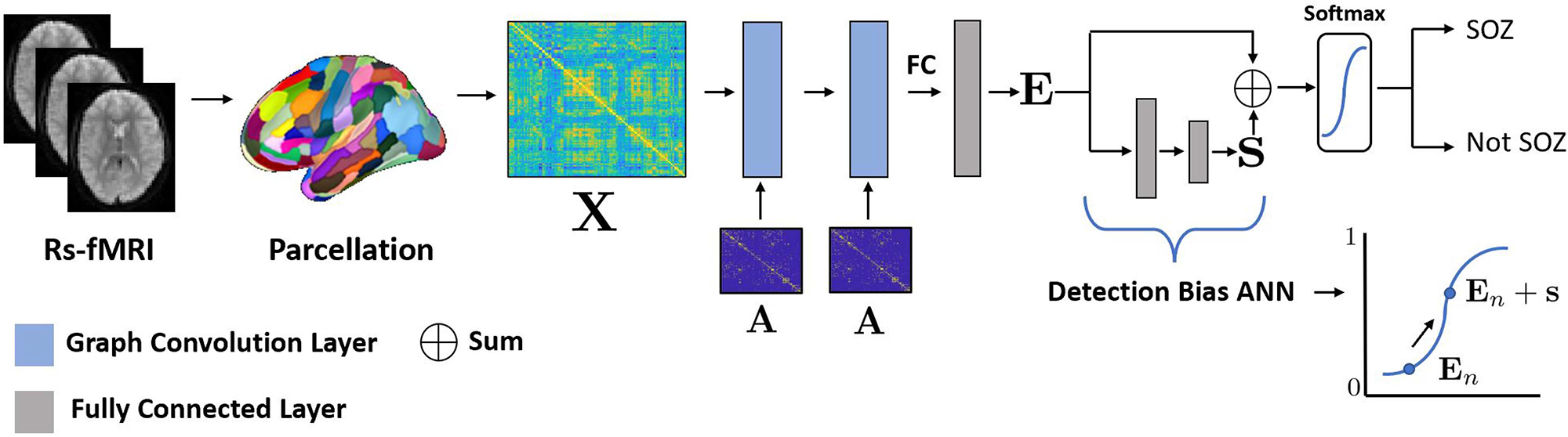
The overview of our model schematic. First, we apply a parcellation to the rs-fMRI and construct the subject specific functional connectivity information **X**. Our network contains two graph convolution layers which include the adjacency matrix **A**. The network uses an artificial neural network (ANN) for node classification. To improve detection of the EZ class, we added a separate ANN to learn a subject-specific bias term **s**, which is added to the node-wise predictions **E**. Our model classifies each ROI from the parcellation as either belonging to the EZ or not.

DeepEZ is designed under the assumption that there are subtle but widespread connectivity patterns associated with the EZ. Inspired by the rs-fMRI literature, we use a weighted similarity matrix to capture whole-brain rs-fMRI connectivity [46], [47], [54]. Our DeepEZ architecture exploits the topological properties of rs-fMRI connectivity data via a set of graph convolutions. To integrate biological knowledge, we use structural connectivity, derived from d-MRI tractography, to define the underlying graph, thus emphasizing signal propagation along anatomical pathways. DeepEZ includes a subject-specific detection bias to account for patient differences and improve generalizability. Finally, we incorporate a clinically relevant asymmetry term into our loss function to provide crucial lateralization information. Fig. 2 provides a graphical overview of our DeepEZ framework.

#### 1) Graph Convolution Network:

Formally, let *N* be the number of brain regions in our parcellation and *T* be the number of time points for a rs-fMRI scan. We define $m_{i}\in {\mathbb{R}}^{T\times 1}$ as the average time series extracted from region *i*, normalized to have zero mean and unit variance. Following the work of [54], we construct the input functional connectivity matrix as follows:

(1) $$X=\exp\left[M^{T}M-1\right]$$

where $M\in {\mathbb{R}}^{T\times N}$ aggregates the region-wise time series **m**_*i*_ as columns. (1) ensures non-negative input values, such that anti-correlated regions have connectivity close to zero, and highly correlated regions have connectivity close to one.

As shown in Fig. 2, DeepEZ processes the input data via two spatial graph convolutions. Let $A\in {\mathbb{R}}^{N\times N}$ be a binary adjacency matrix used for spatial graph filtering [86]. As described in the previous section, we use d-MRI tractography to construct **A**. In this case, an entry **A**_*ij*_ = 1 denotes an anatomical pathway connecting regions *i* and *j*. Each graph convolution produces an activation map $H_{l}\in {\mathbb{R}}^{N\times F_{l}}$, where*l* ∈ {1,2} denotes the layer number. The learnable parameters in each graph convolution are a weight matrix $W_{l}\in {\mathbb{R}}^{F_{l}\times F_{l+1}}$ and a constant bias $b_{l}\in {\mathbb{R}}^{1\times F_{l+1}}$. The activation maps are generated via the layer propagation rule:

(2) $$H_{1}=\phi \left(AXW_{1}+b_{1}\right)$$

(3) $$H_{2}=\phi \left(AH_{1}W_{2}+b_{2}\right)$$

The multiplication by **A** in (2)–(3) aggregates the region-wise representation based on their direct neighborhood [86].

#### 2) Subject-Specific Detection Bias:

We treat EZ identification as a two-class classification problem, where each region *i* classified as either belonging to the EZ or not. Here, the output **H**_2_ of our GCN cascade is fed through a fully-connected layer to obtain $E\in {\mathbb{R}}^{N\times 2}$

(4) $$E=\phi \left(H_{2}W_{fc}\right).$$

Similar to the graph convolutions, the weight matrix $W_{fc}\in {\mathbb{R}}^{F_{2}\times 2}$ is learned during training.

One challenge with our clinical rs-fMRI dataset is heterogeneity, both in the EZ locations and in the data acquisition procedures (e.g., scanner type). To improve detection of the EZ class, we introduce a novel concept known as subject-specific detection bias (SSDB), which helps to mitigate variation in the input data distributions. The SSDB $s\in {\mathbb{R}}^{1\times 2}$ is learned via a simple 2-layer artificial neural network (ANN) and is added to each row of **E** to obtain the final predictions. Mathematically, let {**G**_*l*_, **c**_*l*_} denote the weight matrix and constant offset for each layer *l* ∈ {1, 2} of the ANN. Our subject-specific bias term **s** is computed as follows:

(5) $$s=\phi \left(G_{2}\phi \left(G_{1}E+c_{1}\right)+c_{2}\right).$$

An illustration of the effect of the SSDB on predicting whether a region *n* belongs to the EZ class is shown in the bottom right of Fig. 2. Empirically, we observe that the bias improves the sensitivity of detecting the EZ class. Following the SSDB addition, a softmax function is applied and each region is classified as belonging to the EZ or not using a max operator.

#### 3) EZ Classification Via Weighted Class Prediction and Contralateral Loss Function:

There exists a large class imbalance in our dataset, as on average 7.3 % of the regions lie within the resection boundary that denotes the EZ. Since the GCN layers are designed to operate upon a whole-brain connectivity matrix, traditional data augmentation techniques would not solve our class imbalance problem. Following the work of [55], [56], we train our model with a modified Risk-Sensitive Cross-Entropy loss function [74], which is designed to handle a class membership imbalance. Formally, let *δ*_*i*_ be the risk associated with class *i*. If *δ*_*i*_ is large, then we pay a larger penalty for misclassifying samples belonging to class *i*.

Beyond the class imbalance, it has been shown that contralateral areas of the brain have high rs-fMRI correlation [37], [64], often causing them to be treated similarly in downstream analyses. In contrast, we expect the EZ to be lateralized [14], [36]. We leverage this asymmetry in the second term of our DeepEZ loss function by specifying that regions contralateral to the predicted EZ should be classified as normal.

Let *N*_*e*_ denote the nodes that belong to the EZ (labeled without loss of generality as class #2), and let *c*(*n*) denote the contralateral counterpart to region *n*. Our training loss function consists of the following two terms:

(6) $$ℒ=-\underset{\text{Weighted}\;\text{Cross}\;\text{Entropy}}{\underset{︸}{\sum_{n=1}^{N}\sum_{i=1}^{2}\delta_{i}y_{n,i}\log{\hat{y}}_{n,i}}}-\lambda \underset{\text{EZ}\;\text{Contralateral}\;\text{Term}}{\underset{︸}{\frac{1}{N_{e}}\sum_{n\in N_{e}}\left({\hat{y}}_{n,2}-{\hat{y}}_{c(n),2}\right)}}.$$

The quantities ${\hat{y}}_{n,i}$ in (6) denote the DeepEZ prediction for the baseline (*i* = 1) and EZ (*i* = 2) classes at each region *n*. As seen, the first term of (6) accounts for the class imbalance, and the second term enforces hemispheric asymmetry in the final EZ predictions. Finally, λ balances the contributions of the two loss terms.

#### 4) Implementation Details:

We implement DeepEZ in Py-Torch [57] using the Adam optimizer with weight decay (*wd*) and *∈* for regularization. The LeakyReLU (*x*) = max (0*, x*) + 0.1· min(0*, x*) activation function is applied at each hidden layer of the network in Fig. 2. A softmax activation is applied at the final layer for region-wise classification.

To prevent undue bias, we tune the hyperparameters *δ*_1_*, δ*_2_, λ in (6) and the Adam optimization routine based on 50 subjects drawn from the HCP dataset. Specifically, we randomly selected a portion of the brain regions in each subject to denote an “artificial EZ ”. We then use 10-fold cross validation to fix the hyperparameters used in all experiments. For *δ*_1_ and *δ*_2_, we performed a coarse grid search from 0 – 10 in increments of of 10^−1^ until we found a suitable range of performance. We then performed a finer grid search in increments of 10^−2^. For the parameter λ, we performed a fine grid search over 0 – 1 in increments of 10^−3^. Table II reports the resulting parameter values for our final DeepEZ implementation.

### C. Baseline Methods

We compare DeepEZ against three competing methods from the literature and seven ablated versions of our model:

- ICA approach of [36] (ICA1)
- ICA approach of [85] (ICA2)
- The BrainNetCNN [43] adopted for region-wise classification (BN-CNN)
- Ablation #1: No SCT and No SSDB (GCN)
- Ablation #2: No SSDB (GCN-SCT)
- Ablation #3: No SCT (GCN-SSDB)
- Ablation #4: DeepEZ with an identity matrix replacing **A** (GCN-I)
- Ablation #5: DeepEZ with patient-specific d-MRI matrices replacing **A** (GCN-**A**_*subj*_)
- Ablation #6: DeepEZ with a randomly sampled matrix replacing **A** (GCN-**A**_*rand*_)
- Ablation #7: DeepEZ with topology preserved matrix replacing **A** (GCN-**A**_*top*_)

The first baseline is a traditional machine learning approach for EZ localization described in [36] Specifically, the ICA method extracts features from each independent component (IC) and then classifies each IC as belonging to the EZ or not via a linear support vector machine. The IC farthest from the boundary is selected as the final EZ for that patient. We chose this ICA baseline because it performed the best in the meta-analysis of [15], which compares seven different ICA methods for EZ localization using rs-fMRI. The second baseline described in [85] employs a screening process where ICs are sequentially discarded based on rules such as contralateral correlation and power spectrum density and the remaining ICs are considered belonging to the EZ class. We note that the original method is not fully automated. For example, visual inspection was used on a subject that had multiple independent components (ICs) that survived the rule-based screening process. In an effort to provide fair comparison across methods, we automate the work of [85] by combining the predictions of any ICs that pass the rule-based screening.

The third baseline is a modified version of the BrainNetCNN model developed in [43], which was originally designed to regress cognitive scores from structural connectivity matrices. The BrainNetCNN architecture uses cross-shaped convolutional filters to leverage topological relationships in connectivity data. We have modified the final layers of the original architecture to perform region-wise classification input rather than patient-level phenotypic prediction.

Our next three baselines focus on the EZ contralateral term (SCT) and the subject specific detection bias (SSDB) in our DeepEZ framework. As seen, we systematically ablate the components to determine the performance gain derived from each one. The last four baselines use the same architecture and loss function but vary the anatomical connectivity matrix **A** in the spatial graph convolutions. The GCN-I baseline replaces **A** with the identity matrix, effectively removing the anatomical regularization from our model. The GCN-**A**_subj_ baseline replaces **A** with personalized graphs computed from the patient-specific d-MRI. Due to variations in the data and tractography outputs, the edges in **A**_subj_ vary across patients, i.e., the graphs are slightly mismatched. The GCN-**A**_rand_ baseline replaces **A** with a randomly-sampled symmetric matrix **A**_rand_. Finally, the GCN-**A**_top_ baseline replaces **A** with a matrix that reflects the same geometric topology as **A**. To obtain **A**_top_, we first bin the edge weights from the unthresholded version of **A** and then randomly shuffle edges within each bin [62]. We then threshold to obtain **A**_top_. Similar to the proposed model, we kept **A**_rand_ and **A**_top_ fixed across patients within each CV fold. For robustness, we run a repeated 7-fold CV procedure for all methods, and we sample the matrix **A**_rand_ 50 times and report the average statistics.

## III. Experiments and Results

### A. EZ Detection Performance

**Fig. 3.**
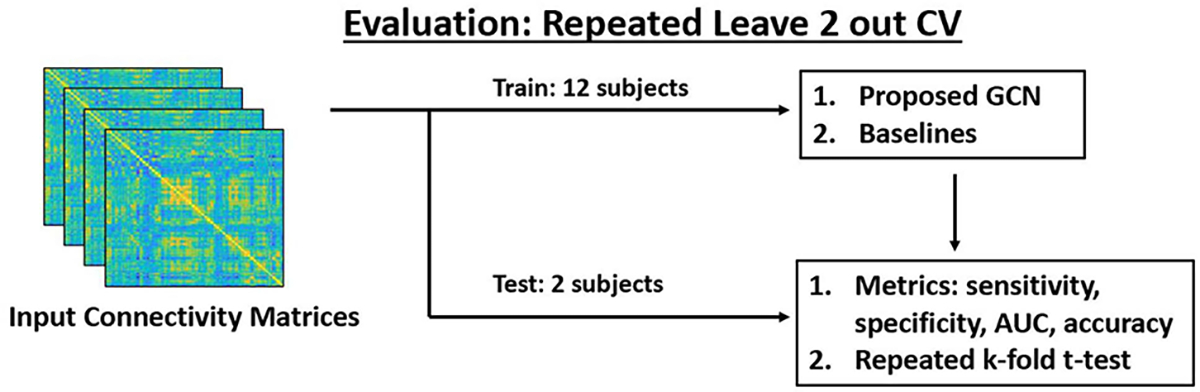
We use repeated 7-fold CV for model training and testing. We report the mean and standard deviation of the sensitivity, specificity, AUC, and accuracy across runs. For each baseline, we report the FDR corrected p-value that DeepEZ achieves significantly higher AUC.

We evaluate the performance of each method using a repeated 7-fold CV setup, where each fold contains 2 subjects and each repeated sampling (i.e., run) ensures different fold membership. Fig. 3 shows the evaluation workflow of our experiments. We report the mean and standard deviation performance across 90 unique runs for the following metrics: sensitivity (TPR), specificity (TNR), accuracy, and area under the receiver operating characteristic curve (AUC). To demonstrate a statistically significant performance gain, we perform a t-test on the AUC metric comparing each baseline with DeepEZ. The test statistic corrects for dependencies between the resampled folds, as outlined in [12]. Using this statistic, we compute a p-value and apply FDR correction. Since the ICA2 method is based on deterministic rules and not learned by a classifier, the results are the same across CV folds. Thus, we report only a single average across patients for each metric, as opposed to a mean±standard deviation. We use a one-sample t-test to determine statistical significance for ICA2.

Table III summarizes the EZ detection performance for each method. We observe that DeepEZ achieves the highest sensitivity, precision, F1 and AUC. While the specificity is slightly lower than the GCN-**A**_*X*_ baselines that swap out the HCP matrix **A**, the performances are within a standard deviation. Minor variations in accuracy can also be attributed to the class imbalance between EZ and non- EZ regions. The performance gains of DeepEZ are underscored by the AUC t-test, where we observe a statistically significant (*p* < 0.05) improvement for DeepEZ over all baseline methods except for GCN-**A**_top_ method. This result highlights that the performance gain of DeepEZ can be largely attributed to the graph topology (e.g., small-world connectivity) when fusing the rs-fMRI connectivity information across layers.

We note that the competing ICA1 and BN-CNN methods are not well-suited to the task, possibly due to the heterogeneity of our clinical cohort. While ICA2 performs much better than ICA1, it cannot match the performance of DeepEZ. One issue with the rule-based ICA methods is that the selection criteria on one dataset may not generalize well to another. While an end-to-end model such as DeepEZ can easily be retrained on new data, modifying a rule-based approach is nontrivial. The ablated models perform slightly better than ICA1, ICA2 and BN-CNN, but still not on par with DeepEZ. In fact, we observe a notable performance gain using the SSDB, which suggests that a subject-specific approach may be useful to overcome heterogeneity in clinical prediction tasks. There is a similar performance gain when incorporating the contralateral loss term (SCT), which emphasizes the asymmetry associated with our problem. We observe a marked decline in sensitivity when replacing the d-MRI connectivity matrix **A** with identity. This suggests that using information about anatomical pathways is crucial for EZ localization. Interestingly, we also note a performance decline when using the patient-specific d-MRI information encoded in **A**_subj_. We hypothesize that this is due to the inconsistency of the edges across patients, particularly in our small dataset. This hypothesis is supported by the results for GCN-**A**_rand_ and GCN-A_top_, which performs slightly better than GCN-**A**_subj_. Recall that, while random, the graphs in GCN-**A**_rand_ and GCN-**A**_top_ are fixed. We find that model training is more stable when the same **A** matrix is used for each patient. Finally, GCN-**A**_top_ performs the best out of the baselines, implying a benefit to using topological information.

**Fig. 4.**
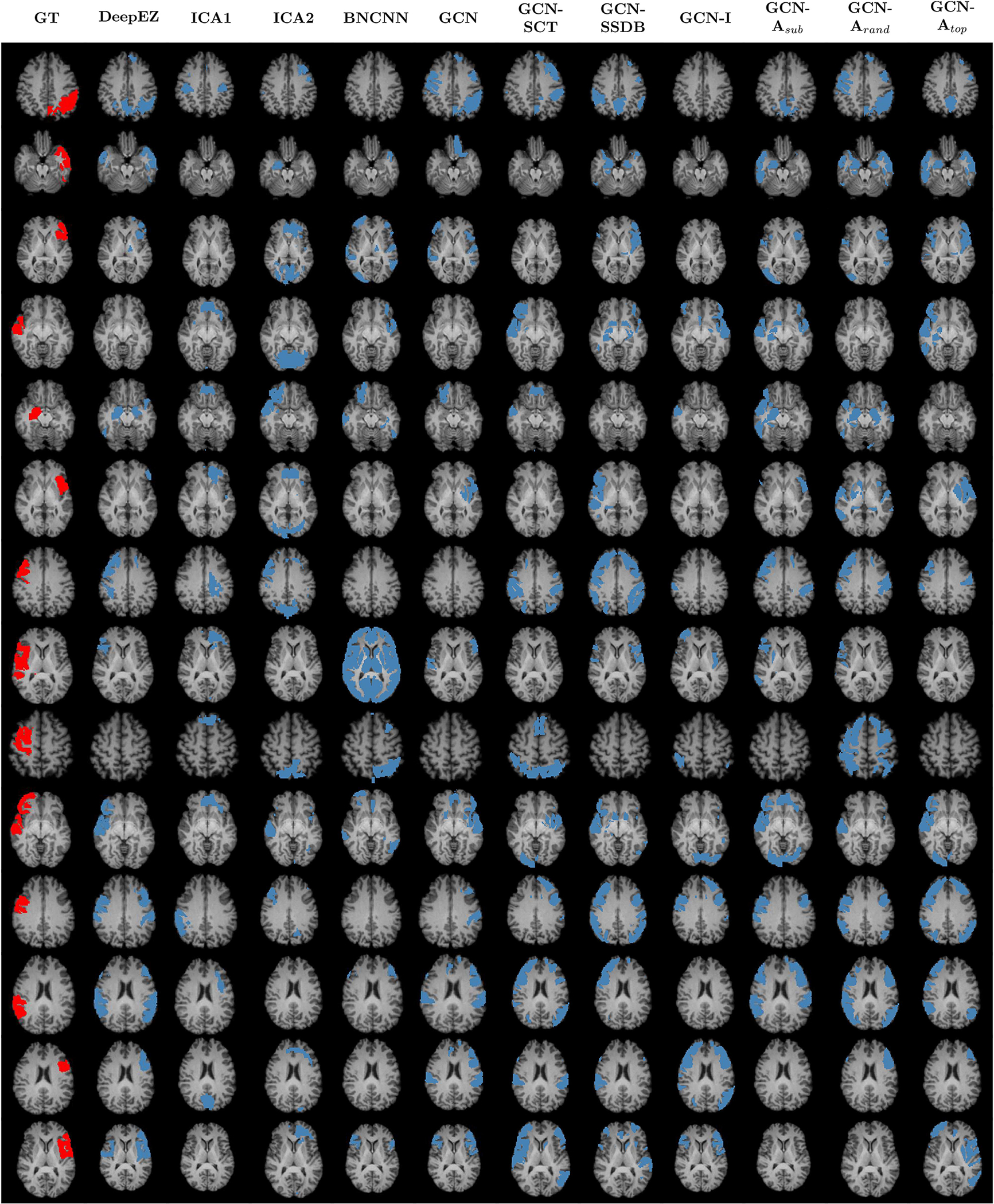
Axial view of ground truth (red) and model predictions (blue) for all patients in the UW Madison dataset. Each row corresponds to a single patient, organized from 1–14 according to Table I. Model names are displayed at the top of each column.

Fig. 4 illustrates axial views of the ground truth (red) and predicted (blue) labels for all 14 patients across each method considered. Each row represents a patient (numbered 1–14 in Table I) and each column represents one method. As shown, DeepEZ localizes correct regions in most patients while omitting non-EZ regions. An example of this can be see for Patient 1 (first row), where DeepEZ aligns well with the ground truth labels, while avoiding the spurious predictions by the GCN-X methods. Furthermore, DeepEZ is the only method to localize the resections in Patient 3 and Patient 10, while not incurring incorrect contralateral predictions. We observe that the ICA and BNCNN methods are poorly suited for the task and rarely produce correct predictions. Overall, we observe similar predictions made by the DeepEZ and GCN-**A**_top_ methods, likely due to the preserved network topology. Overall, we observe that DeepEZ achieves a good balance between correct localization while avoiding false positives.

**Fig. 5.**
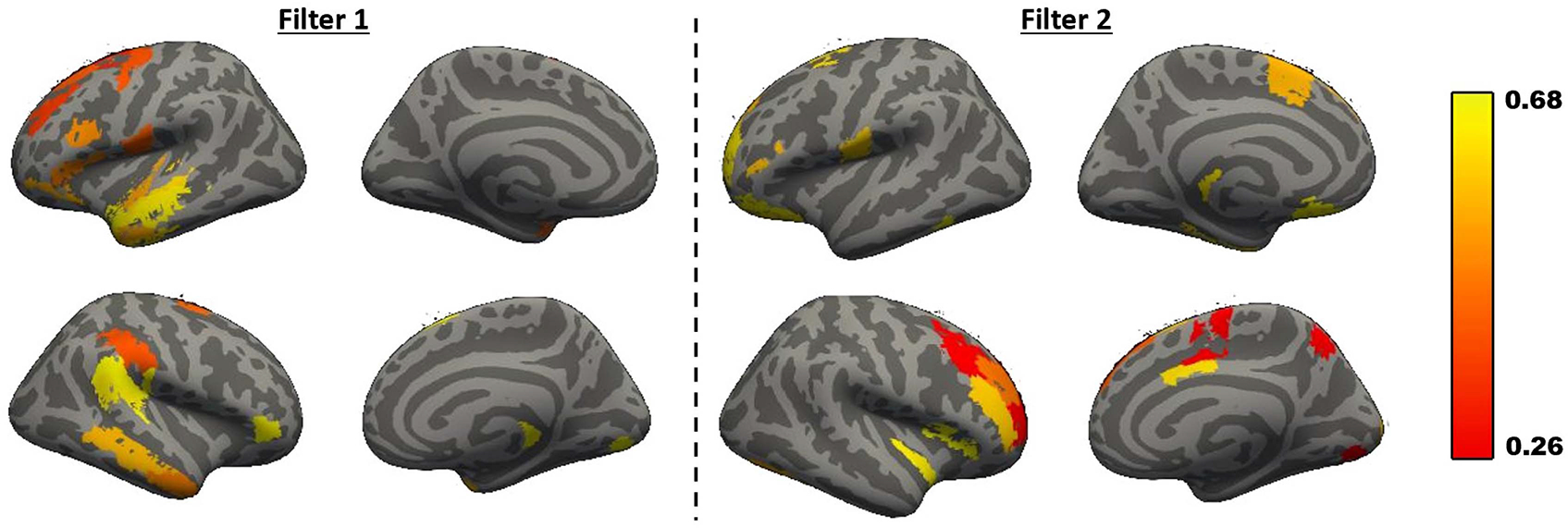
Regions implicated by the two most commonly learned convolutional filters in DeepEZ. One filter (**L**) identifies the temporal lobe while the other (**R**) identifies the frontal regions. The distribution of these regions mimic the EZ labels in our dataset.

Finally, we visualize the most commonly learned graph convolutional filters of DeepEZ. Accordingly, we extracted the first-stage graph convolution weight matrices $W_{1}\in {\mathbb{R}}^{246\times 120}$ learned during each of our repeated 7-fold CV runs. Each column of **W**_1_ represents a different convolutional filter that can be visualized by plotting the magnitude of the weights back on to the brain. We first aligned the columns of **W**_1_ across the repeated CV folds using a Procrustes algorithm and then masked each column to identify the top ten regions implicated by that filter. We identified two activation patterns that were consistently learned by DeepEZ. Fig. 5 shows these filters projected onto the cortical surface, where the color denotes the average activation across repeated CV folds.

We note that one filter (left) implicates regions within the temporal lobe while the other filter (right) implicates the regions associated with the frontal lobe. These patterns mimic the distribution of EZ labels in our UW Madison dataset. Thus, in a data-driven manner, DeepEZ focuses its analysis on stereotypical patterns associated with the EZ.

### B. Assessing Model Robustness

In this section, we assess the robustness of DeepEZ to four aspects of our experimental setup: (1) the choice of brain parcellation, (2) the number of GCN layers, (3) the relative weighting *δ*_2_ for the EZ class in (6), and (4) the small dataset size used for model training.

#### 1) Varying Parcellation Choice:

It has been shown that the choice of parcellation can have a tremendous impact on rs-fMRI analyses [21], [48]. For example, coarse parcellations mitigate the effects of noise but can blur subtle effects, whereas fine parcellations preserve detailed phenomena but can be overwhelmed by environmental confounds. In addition, the Brainnetome atlas (BNA) used in Table III is symmetric where each region has a direct contralateral region, which is not the case with all parcellations. To explore this, we apply DeepEZ using three different scales of the Craddocks functional parcellation [18]. The Craddocks atlas was derived using a spectral clustering algorithm on the rs-fMRI data from healthy subjects. The different scales come from varying the number of clusters. In this work, we use the *N* = 178*, N* = 318, and *N* = 384 scales, which include both coarser and finer parcellations than the BNA *N* = 246 atlas to assess the effect that resolution has on performance. Once again, we use repeated 7-fold CV to quantify performance.

Table IV reports the accuracy and AUC when applying DeepEZ to each of the parcellations defined above. The p-values are computed with respect to the original BNA atlas. To account for the fact that regions in the Craddocks atlases are not symmetrically defined across the hemispheres, our SCT loss function considers the region with centroid closest to the contralateral location as the counterpart *c*(*n*) in (6). Based on a *p* < 0.05 threshold, we only observe a significant performance difference in AUC with the CC-178 atlas. This observation suggests that a finer parcellation is better suited for EZ localization. In contrast, DeepEZ is robust using either the CC-318, CC-384, or BNA atlas, which suggests model stability across different parcellations.

#### 2) Number of GCN Layers:

At a high level, the GCN layers of DeepEZ perform a random walk on the brain graph defined by the anatomical connections in d-MRI. Our choice of two GCN layers in DeepEZ can analyze the rs-fMRI connectivity patterns associated with path lengths ≤ 2 but cannot capture higher-order information. To probe this effect, we conduct a robustness experiment in which we vary the number of GCN layers in DeepEZ and use the repeated 7-fold CV strategy in Fig. 3 to quantify the performance of each method.

Table V reports the performance across 1–4 GCN layers. We observe that the proposed architecture (2 GCN layers) achieves the best trade-off between true positive and false positive detections, as quantified via a t-test on the AUC. There are two interpretations for this result. First, it appears that the rs-fMRI connectivity patterns associated with the EZ are the most prominent at a walk of length of two, with diminishing returns beyond this point. Second, increasing the number of GCN layers also increases the number of model parameters, which may lead to overfitting. Taken together, we believe that two GCN layers balances the trade-off between capturing discriminative patterns without overfitting on small datasets.

#### 3) EZ Detection Hyperparameter:

**Fig. 6.**
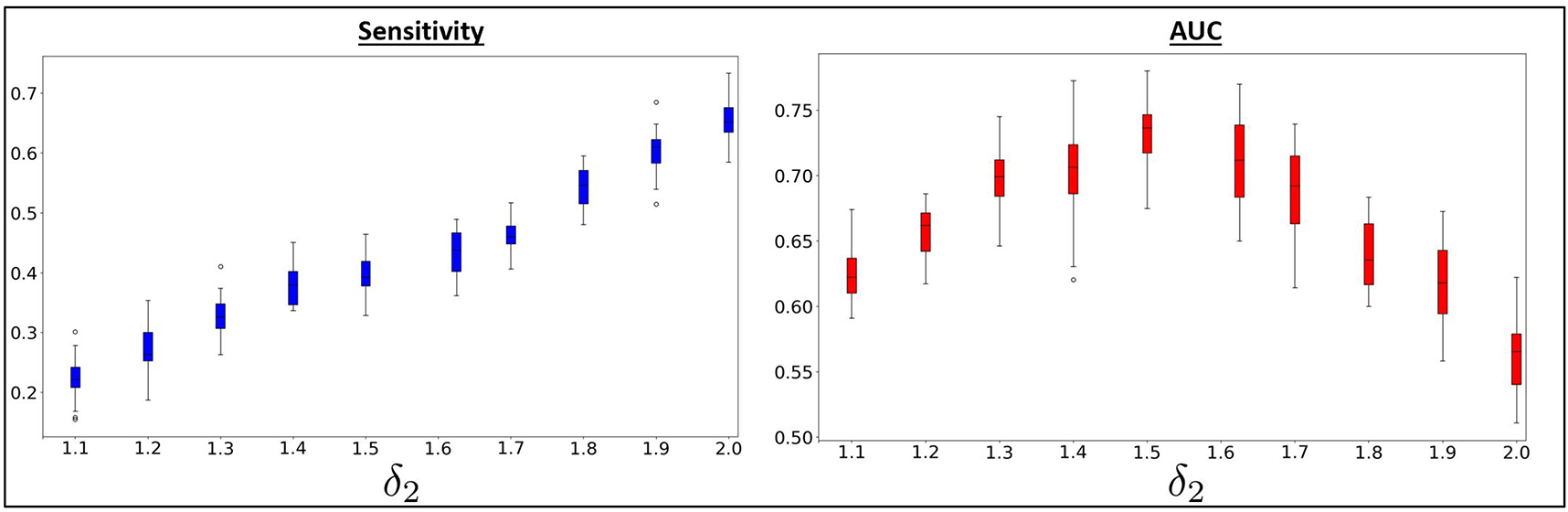
Sensitivity (left) and AUC (right) for proposed method while sweeping *δ*_2_ in increments of 0.1. We observe as *δ*_2_ increases, sensitivity increases, but AUC eventually decreases.

One of the key aspects of DeepEZ is the weighted cross-entropy loss to handle class imbalance. To probe this effect, we sweep the EZ detection hyperparameter *δ*_2_ in in increments of 0.1 while keeping the other hyperparameters fixed. Fig. 6 shows the sensitivity (left) and AUC (right) metrics as *δ*_2_ varies over the range [1.1,2.0]. As expected, sensitivity increases with *δ*_2_ due to the higher penalty for incorrectly classifying EZ regions as baseline. However, the AUC metric peaks at 1.5 and steadily decreases, which suggests that DeepEZ incurs too many false positives at larger values of *δ*_2_.We observe relatively stable performance in both metrics over the range *δ*_2_ = [1.4 – 1.6]. Finally, we note that the weighted cross-entropy loss is useful from a clinical perspective, as it is more important not to miss the EZ regions at this stage of therapeutic planning for epilepsy.

#### 4) Training Data Augmentation:

Data augmentation has been shown to improve the performance of deep learning models due to providing more information about the underlying data distribution [59], [61]. Given the small sample size (*N* = 14), we explore whether DeepEZ would benefit from data augmentation. Here, we sub-sampled the time series data using a continuous sliding window to create 10 distinct new training similarity matrices for each subject. Our augmented dataset contains an order of magnitude more data points (*N* = 140). Our evaluation strategy remained the same as depicted in Fig. 3. Table VI reports the EZ detection performance for DeepEZ with and without data augmentation. We observe a small performance boost in each metric and smaller standard deviations when using data augmentation during training. However, we note that the performance gain in AUC is not statistically significant, as indicated in the last column of Table VI. This result demonstrates that DeepEZ is able to effectively mine the information present in our original dataset for generalizable EZ localization.

## IV. Discussion

We have introduced DeepEZ, a novel deep learning approach for EZ localization based on rs-fMRI connectivity. DeepEZ relies on spatial graph convolutions that leverage biologically-inspired anatomical pathways to aggregate neighborhood information during forward propagation. These graph convolutions are complemented with a subject-specific detection bias (SSDB) to mitigate inter-patient differences in connectivity and an asymmetry loss term to encourage lateralized predictions. In comparison to baseline methods, DeepEZ achieves statistically improved AUC for detecting EZ regions. Via ablation studies, we show that these performance gains are linked to the EZ contralateral term (SCT) and the SSDB. In subsequent anaylses, we demonstrate that DeepEZ is robust to varying the parcellation used for analysis and performs comparably with and without data augmentation. We also show that DeepEZ achieves robust performance within a range of *δ*_2_ in the weighted cross-entropy loss term.

Clinical rs-fMRI studies often lack statistical power due to small sample sizes [1] with logistical constraints making it difficult to acquire additional data for analysis. In our case, the UW Madison dataset contains a specialized cohort of pediatric focal epilepsy patients who underwent surgical resection of the EZ. Currently, rs-fMRI is not a commonly acquired modality for epilepsy patients, which limits our ability to grow the dataset further. Thus, to maximize the sample size for model training and evaluation, we include patients that have their scans taken from two different scanners (1.5 T and 3 T in Table I) which is common in the literature [49], [70], [77]. Accordingly, we have designed DeepEZ to mine information from smaller heterogeneous datasets. Relevant attributes include a relatively small number of learnable parameters, a biologically informed spatial graph, the SSDB module to improve sensitivity, and a SCT to encourage clinically relevant EZ localization patterns. We demonstrate state-of-the-art in Section 3, along with a robustness to different modeling choices and data augmentation. The fact that DeepEZ harmonizes information across scanners is particularly encouraging, as inter-scanner differences are known to confound deep learning models [39]. Overall, these results increase our confidence in DeepEZ as a prospective clinical tool for epilepsy evaluation.

Perhaps the most interesting result of this work is that using subject-specific anatomical connectivity information does not improve localization performance. In fact, the sensitivity, precision, F1 and AUC are worse than when the graph **A** is fixed according to the normative HCP dataset. There are three different facets to this result. From an imaging standpoint, there is more variability in our UW Madison dataset due to scanner differences (e.g., 1.5 T versus 3 T), patient age (9–18 years), and heterogeneous pathologies. This variability may lead to “incorrect” tractography outputs, as compared to the underlying neurophysiology. In contrast, the HCP acquisition sequences have been carefully validated on a standardized cohort, and the preprocessing pipelines have been optimized for the data. Consequently, the matrices **A** may reflect long-range and distributed anatomical pathways more accurately than **A**_subj_. From an optimization standpoint, the edges are inconsistent across the patient-specific matrices **A**_subj_. This inconsistency can lead to instability during training, particularly given the small sample size (*S* = 14). Future work will include comparing the influence of **A** and **A**_subj_ as the dataset grows in size. Finally, from a neurobiological standpoint, our robustness study in Section III-B suggests that two GCN layers is optimal for EZ localization. Thus, DeepEZ only fuses information across two-stage pathways. Given that we operate at the region level, the HCP template may be sufficient for this operation without needing patient-specific connectivity.

In line with the above observation, our experiments demonstrate that replacing the anatomical connectivity matrix with **A**_top_ achieves similar performance. This result underscores the importance of *network topology* over individual anatomical connections. It also suggests that DeepEZ is robust to variations in the anatomical connectivity matrix used for **A**. Thus, we conclude that acquiring rs-fMRI data alone is sufficient for EZ localization, which reduces the logistical burden of integrating DeepEZ into the clinical workflow.

We note that there is considerable prior work that uses ICA for EZ localization in rs-fMRI [11], [32], [36]. The meta-analysis of [15] determined that among these, the machine learning approach of [36] (baseline 1 in this work) achieves the best odds ratio. However, as reported in Table III, this method fails to localize the EZ for our cohort. One possible reason is that the handcrafted features chosen in [36] may not generalize well to different cohorts. Another drawback of this method is that it selects just one independent component as the EZ, when there might exist multiple epileptic sources across overlapping components [35]. In fact, we observed in our experiments that the independent components did not overlap well with the surgical resection boundaries used as the pseudo ground truth EZ. We also note that since this method performs ICA at the subject level, there is substantial variability in the component locations across patients. In contrast, DeepEZ uses a well-defined functional parcellation, which allows for both a fine resolution analysis and group-level concordance in the region definitions. We also demonstrate that DeepEZ is robust to the choice of parcellation, which gives the user more flexibility when applying the framework to clinical data.

Accurate EZ localization through noninvasive imaging can significantly impact epilepsy treatment. Concordance between individual modalities (EEG, structural MRI) improves the prognostic yield of first tier evaluation of patients with epilepsy. In addition, the odds of seizure freedom following surgical treatment in medication refractory epilepsy are associated with correct identification of an epileptogenic lesion [40]. Hence, in non-lesional cases, successful delineation of a EZ based on rs-fMRI connectivity can improve treatment outcomes by better identifying surgical resection targets [22]. One limitation of this paper is that, as our data was acquired by the UW Madision health system during routine preoperative evaluation, we do not have access to healthy controls or multi-focal epilepsy patients (who would not be considered surgical candidates) for model training. However, acquiring and integrating this data into DeepEZ remains a crucial direction of future work. Finally, we emphasize that our deep learning framework can be adapted to other applications, such as preoperative mapping of the eloquent cortex [54]–[56].

## V. Conclusion

We have introduced a novel deep learning based method to localize the seizure onset zone in focal epilepsy patients. Our DeepEZ framework combines the representational power of deep neural networks with domain specific modeling choices, such as asymmetry of the EZ and anatomical regularization. We show that DeepEZ outperforms an existing ICA method from the literature and various deep learning baselines in localizing the EZ. We also demonstrate the robustness of our framework to various modeling choices, such as parcellation and hyperparameter choice. Our results provide evidence for EZ identification in epilepsy using noninvasive rs-fMRI based imaging that has the potential to improve the diagnostic work up and therapeutic planning in treatment of epilepsy.

## Supplementary Material

supp1-3187942

## Figures and Tables

**TABLE I:** Demographic Information, EZ Location, and Scanner Type, and Outcome for Each Patient in the UW Madison Dataset

Subject	Age	Gender	EZ Location	Scanner	Seizure Control
1	14	M	left anterior and medial parietal	3T	Engel IA, ILAE 1
2	17	M	left anterior temporal lobe	3T	Engel IA, ILAE 1
3	10	F	left frontal pole	3T	Engel IA, ILAE 1
4	11	M	right temporal lobe	3T	Engel IA, ILAE 1
5	15	F	right anterior frontal/amygdala	3T	Engel IIIA, ILAE 4
6	11	M	left inferior frontal operculum and insula	3T	Engel IA, ILAE 1
7	13	F	right middle frontal region	3T	Engel IIIA, ILAE 2
8	14	M	right posterior temporal and parietal region	1.5T	Engel IA, ILAE 1
9	15	F	right anterior precentral gyrus	1.5T	Engel IIIA, ILAE 4
10	18	M	right temporal	3T	Engel IIB ILAE 2
11	9	M	right middle frontal lobe	1.5T	Engel IA, ILAE 1
12	15	M	right middle parietal lobe	1.5T	Engel IVA, ILAE 5
13	16	M	Left inferior frontal	3T	Engel IIIA, ILAE 4
14	11	M	left inferior frontal gyrus	3T	Engel IA, ILAE 1

**TABLE II:** Hyperparameters Determined Via Cross Validation on a Separate Cohort Drawn from the HCP Dataset

Parameter	Value	Parameter	Value

(*δ*_1_, *δ*_2_)	(0.29, 1.52)	λ	0.017
*lr*	0.005	Epochs	200
*ϵ*	1 × 10^−8^	*wd*	5 × 10^−5^

**TABLE III:** Mean Plus or Minus Standard Deviation for Sensitivity, Specificity, Precision, F1-Score, Accuracy, and AUC, The T-Score Compares the AUC of DeepEZ with Each Baseline; We Also Note the Corresponding FDR Corrected P-Value. We Use a Two-Sample T-Test Based on the Repeated 7-Fold CV For All Models, Except ICA2; Here, We Use a One-Sample T-Test

Method	Sensitivity	Specificity	Precision	F1	Accuracy	AUC	t-score	p-value

ICA1	0.071 ± 0.034	0.78 ± 0.045	0.09 ± 0.035	0.08 ± 0.029	0.57 ±0.033	0.52 ± 0.023	22.26	< 10^−10^
ICA2	0.25	0.7	0.31	0.28	0.69	0.6	7.13	< 10^−10^
BN-CNN	0.099 ± 0.048	0.72 ± 0.036	0.11 ±0.046	0.11 ±0.027	0.78 ±0.015	0.56 ± 0.035	11.84	< 10^−10^
GCN	0.17 ±0.051	0.78 ± 0.032	0.26 ± 0.039	0.20 ± 0.038	0.81 ± 0.032	0.62 ± 0.035	7.66	< 10^−10^
GCN-SCT	0.22 ± 0.059	0.81 ±0.036	0.29 ± 0.042	0.25 ± 0.041	0.83 ± 0.029	0.65 ± 0.038	5.13	8.6 × 10^−7^
GCN-SSDB	0.31 ±0.056	0.83 ±0.031	0.43 ± 0.048	0.36 ± 0.039	0.85 ± 0.03	0.70 ± 0.034	2.15	0.023
GCN-I	0.27 ±0.061	0.86 ± 0.034	0.39 ± 0.037	0.31 ± 0.028	0.87 ±0.041	0.68 ± 0.033	3.69	3.3 × 10^−4^
GCS-**A**_*subj*_	0.28 ± 0.046	0.87 ±0.029	0.37 ±0.039	0.32 ± 0.034	0.89 ± 0.031	0.70 ± 0.026	2.03	0.031
GCN-**A**_*rand*_	0.33 ± 0.041	0.86 ±0.031	0.41 ± 0.042	0.37 ±0.037	0.88 ± 0.034	0.71 ± 0.028	1.91	0.046
GCN-**A**_*top*_	0.35 ± 0.039	0.86 ± 0.035	0.47 ± 0.038	0.4 ± 0.035	0.88 ± 0.036	0.72 ±0.019	1.63	0.078
DeepEZ	**0.4 ± 0.044**	0.85 ± 0.033	**0.52 ± 0.039**	**0.45 ± 0.041**	0.88 ± 0.034	**0.73 ± 0.031**		

**TABLE IV:** Mean Plus or Minus Standard Deviation for Accuracy and AUC for Different Parcellations, The Final Column Shows the FDR Corrected P-Values When Comparing the AUCs of the BNA and Craddocks (CC) Parcellations

Atlas	Accuracy	AUC	p-val

BNA-246	0.88 ± 0.034	0.73 ± 0.031	
CC-178	0.85 d= 0.037	0.70 ± 0.032	0.018
CC-318	0.87 ±0.038	0.72 ± 0.039	0.367
CC-384	0.87 ±0.035	0.72 ± 0.031	0.312

**TABLE V:** Localization Performance as the Number of GCN Layers is Varied, the T-Score Compares the AUC of the Proposed DeepEZ With Each Baseline; We Also Note the Corresponding FDR Corrected P-Value

Layers	Sensitivity	Specificity	Precision	FI	Accuracy	AUC	t-score	p-value

1	0.09 ± 0.035	0.93 ± 0.021	0.12 ±0.032	0.1 ±0.029	0.91 ± 0.039	0.55 ± 0.032	12.04	< 10^−10^
2 (Proposed)	**0.4 ± 0.044**	0.85 ± 0.033	**0.52 ±0.039**	**0.45 ± 0.041**	0.88 ± 0.034	**0.73 ± 0.031**		
3	0.27 ± 0.042	0.87 ±0.035	0.38 ± 0.039	0.32 ± 0.037	0.88 ± 0.031	0.70 ± 0.027	2.01	0.039
4	0.33 ± 0.039	0.84 ± 0.029	0.45 ± 0.041	0.38 ± 0.029	0.83 ± 0.028	0.68 ± 0.029	3.6	4.9 × 10^−4^

**TABLE VI:** Mean Plus or Minus Standard Deviation for Sensitivity, Specificity, AUC, and Accuracy With and Without Data Augmentation, the Final Column Shows the FDR Corrected P-Values for the Associated T-Score for Comparing AUC

Augmentation	Sensitivity	Specificity	Precision	FI	Accuracy	AUC	t-score	p-value

Without	0.4 ± 0.044	0.85 ± 0.033	0.52 ± 0.039	0.45 ± 0.041	0.88 ± 0.034	0.73 ± 0.031		
With	0.43 ± 0.033	0.87 ± 0.026			0.89 ±0.015	0.74 ±0.019	−1.28	0.76
