# Supplementary material for: DeepEZ: A Graph Convolutional Network for Automated Epileptogenic Zone Localization From Resting-State fMRI Connectivity
Source: IEEE Trans Biomed Eng. Author manuscript; Available in PMC 2023 Apr 5. (PMC9841829; doi:10.1109/TBME.2022.3187942)
Supplement: supp1-3187942 [file NIHMS1860979-supplement-supp1-3187942.pdf]

Supplementary Results

DeepEZ: A Graph Convolutional Network for Automated Epileptogenic Zone Localization from Resting-state fMRI Connectivity

1 Introduction

This supplement provides further experimental results to augment our main paper. First, we display additional views (axial, sagittal and coronal) of the DeepEZ localization performance in four representative patients. We also show boxplots of the sensitivity and AUC achieved on the entire dataset across all methods considered. Finally, we stratify our patient cohort based on demographic and clinical factors and assess the performance of DeepEZ in each case.

2 Further Visualizations

In this section, we provide further visualizations of our main results.

SFig. 1 shows boxplots of the sensitivity (left) and AUC (right) among all methods. We include this figure to show the spread of performance based on fold membership. As described in the main text, we observe statistically higher sensitivity and AUC for the proposed DeepEZ method.

SFig. 2 shows three views of ground truth (red) and DeepEZ predictions (blue) of four representative patients. Based on clinical evaluation, the EZ

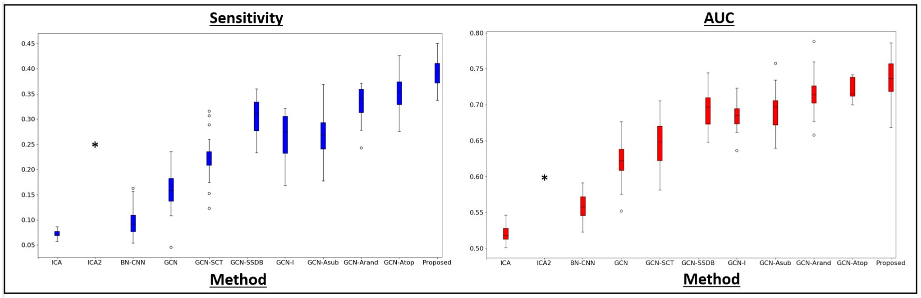

SFigure 1: Boxplots for sensitivity (left) and AUC (right) across each method. The proposed DeepEZ has the best performance across methods considered.

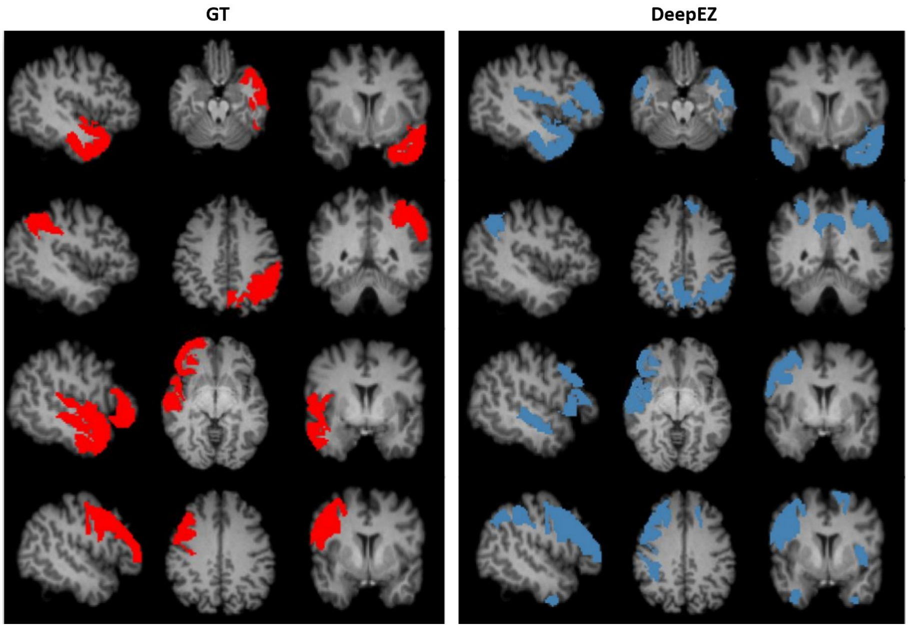

SFigure 2: Ground truth (red) and DeepEZ predictions (blue) for four representative patients. The patients have the EZ located in left temporal, left extra-temporal, right temporal and right extra-temporal from top to bottom respectively.

for these patients are categorized as left temporal, left extra-temporal, right temporal and right extra-temporal, as organized from top to bottom in the figure. We note that DeepEZ can accurately localize the EZ in the second and fourth patients with minimal false positives. For the firth and third patients, DeepEZ exhibits high sensitivity with spurious contralateral predictions.

3 Patient Stratification

It is important to understand how DeepEZ performs based on different clinical and demographic factors, such as patient outcome, gender, and EZ location. We have conducted statistical evaluations based on these factors. Regarding patient outcome, ten patients are seizure free (denoted by either ILAE 1 or ILAE 2)

STable 1: Mean plus or minus standard deviation performance metrics for ILAE 1-2 patients vs ILAE 3-5 using DeepEZ. The t-score compares the AUC between the two, we also note the corresponding FDR corrected p-value.

Outcome	Sensitivity	Specificity	Precision	F1	Accuracy	AUC	t-score	p-value
ILAE 1-2	0.42 ± 0.046	0.85 ± 0.045	0.53 ± 0.048	0.46 ± 0.038	0.87 ± 0.046	0.74 ± 0.039		
ILAE 3-5	0.39 ± 0.045	0.86 ± 0.037	0.47 ± 0.049	0.43 ± 0.041	0.88 ± 0.034	0.73 ± 0.036	-0.34	0.54

and four patients experience seizures but at a reduced rate (Table 1 in main text). STable 1 below shows the performance metrics using DeepEZ for ILAE 1-2 patients and ILAE 3-5 patients. While there is a slightly higher sensitivity and AUC in the seizure free cohort, a t-test on the AUC metric does not indicate significant difference in performance. We further stratified the cohort according to gender, hemisphere, and lobe of the EZ and performed the same t-test. These results are in STables 2-4. Overall, we observe a marginally significant difference in performance between left and right hemisphere SOZ ($p < 0.1$), but this may be due to the small sample size. We do not observe statistical difference in performance across the other stratifications of the cohort. With more data, it would be clinically valuable to see if the model performs statistically better or worse regarding patient outcome and EZ location.

STable 2: Mean plus or minus standard deviation performance metrics for male vs. female patients using DeepEZ. The t-score compares the AUC between the two, we also note the corresponding FDR corrected p-value.

Gender	Sensitivity	Specificity	Precision	F1	Accuracy	AUC	t-score	p-value
Male	0.38 ± 0.046	0.86 ± 0.041	0.49 ± 0.046	0.44 ± 0.039	0.88 ± 0.042	0.73 ± 0.041		
Female	0.41 ± 0.047	0.85 ± 0.039	0.52 ± 0.051	0.45 ± 0.043	0.89 ± 0.039	0.73 ± 0.039	-0.25	0.53

STable 3: Mean plus or minus standard deviation performance metrics for right vs. left hemisphere EZ patients using DeepEZ. The t-score compares the AUC between the two, we also note the corresponding FDR corrected p-value.

Hemisphere	Sensitivity	Specificity	Precision	F1	Accuracy	AUC	t-score	p-value
Right	0.35 ± 0.042	0.84 ± 0.038	0.45 ± 0.046	0.41 ± 0.043	0.87 ± 0.038	0.71 ± 0.039		
Left	0.43 ± 0.043	0.86 ± 0.043	0.55 ± 0.049	0.47 ± 0.042	0.88 ± 0.043	0.74 ± 0.031	1.70	0.064

STable 4: Mean plus or minus standard deviation performance metrics for temporal vs non-temporal EZ patients using DeepEZ. The t-score compares the AUC between the two, we also note the corresponding FDR corrected p-value.

Lobe	Sensitivity	Specificity	Precision	F1	Accuracy	AUC	t-score	p-value
Temporal	0.39 ± 0.049	0.85 ± 0.041	0.5 ± 0.047	0.44 ± 0.045	0.88 ± 0.042	0.72 ± 0.042		
Extra-temporal	0.41 ± 0.042	0.85 ± 0.044	0.52 ± 0.041	0.46 ± 0.041	0.88 ± 0.039	0.73 ± 0.035	0.43	0.41
